# Acquired Resistance to the PRMT5 Inhibitor Confers Collateral Sensitivity to MEK Inhibition in *MTAP*-Null Non-Small Cell Lung Cancer

**DOI:** 10.3390/biom16081198

**Published:** 2026-08-17

**Authors:** Rongjie Fu, Yalong Wang, Ishita Rehman, Ella Bedford, Sana Sharif, Nghi D. Nguyen, Reid T. Powell, Andrew Adams, Weijun Liu, Shuyue Wang, Wei He, Yue Lu, Bin Liu, Pooja Anil Shah, Jordi Rodon Ahnert, Taiping Chen, Weiyi Peng, Clifford C. Stephan, Xinli Liu, Mark T. Bedford, Han Xu

**Affiliations:** 1 Department of Epigenetics and Molecular Carcinogenesis, The University of Texas MD Anderson Cancer Center, Houston, TX 77054, USA; 2 Department of Biotechnology, The Neotia University, Sarisha 743368, West Bengal, India; 3 Department of Pharmacological and Pharmaceutical Sciences, The University of Houston College of Pharmacy, Houston, TX 77204, USA; 4 Texas A&M Health Center for Translational Cancer Research, Houston, TX 77030, USA; 5 Department of Investigational Cancer Therapeutics, The University of Texas MD Anderson Cancer Center, Houston, TX 77030, USA; 6 Department of Biology and Biochemistry, University of Houston, Houston, TX 77004, USA; 7 Department of Bioinformatics and Computational Biology, The University of Texas MD Anderson Cancer Center, Houston, TX 77054, USA

**Keywords:** PRMT5, *MTAP*-null, acquired resistance, MTA-cooperative PRMT5 inhibitor, MAPK signaling, MEK inhibition

## Abstract

Protein arginine methyltransferase 5 (PRMT5) is a synthetic lethal target in methylthioadenosine phosphorylase-deleted (*MTAP*-null) cancers. Second-generation methylthioadenosine (MTA)-cooperative PRMT5 inhibitors preferentially target *MTAP*-null cells while largely sparing *MTAP*-wildtype (*MTAP*-WT) cells, thereby improving tumor selectivity over first-generation PRMT5 inhibitors. Despite encouraging efficacy and safety signals in early clinical studies, the modest objective response rates (ORRs) observed with these inhibitors suggest that intrinsic or acquired resistance may limit their clinical benefit. Here, we investigated acquired resistance to the MTA-cooperative PRMT5 inhibitor BMS-986504/MRTX1719 in *MTAP*-null non-small cell lung cancer (NSCLC) cells and sought to identify therapeutic vulnerabilities that emerge upon resistance. Using multiple in vitro-derived resistant models, we found that acquired resistance was accompanied by cross-resistance to mechanistically distinct PRMT5 inhibitors. Notably, this phenotype was not fully explained by altered PRMT5 activity or changes in MTA levels. High-throughput drug screening of paired sensitive and resistant cells revealed increased sensitivity to MEK inhibitors following acquisition of MRTX1719 resistance in *KRAS*-wildtype NSCLC cells. Consistently, resistant cells exhibited rewired MAPK-related transcriptional programs. Together, these findings identify MEK inhibition as a reproducible collateral vulnerability associated with acquired MRTX1719 resistance in *MTAP*-null NSCLC models and support further evaluation of MEK inhibition as a potential treatment-switching strategy following resistance.

## 1. Introduction

Homozygous deletion of chromosome 9p21.3 is one of the most frequent genomic alterations in human cancers. Among genes mapping to this region, the tumor suppressor cyclin-dependent kinase inhibitor 2A (*CDKN2A*) is the most frequently deleted gene (13.5%), followed by methylthioadenosine phosphorylase (*MTAP*) (9.3%). Because of their close genomic proximity, *MTAP* is commonly co-deleted with *CDKN2A*, with concurrent *MTAP*/*CDKN2A* loss observed in 9.2% of tumors across multiple cancer types, including glioblastoma, mesothelioma, urothelial carcinoma, pancreatic cancer, melanoma, and non-small cell lung cancer (NSCLC) [1,2]. Loss of MTAP leads to accumulation of methylthioadenosine (MTA), which competes with the methyl donor S-adenosylmethionine (SAM) for binding to protein arginine methyltransferase 5 (PRMT5), thereby partially suppressing PRMT5 activity. In *MTAP*-deleted (*MTAP*-null) cancers, this reduction in basal PRMT5 activity creates a therapeutic window for further pharmacologic inhibition of PRMT5 relative to *MTAP*-wildtype (*MTAP*-WT) cells [3,4,5,6,7,8].

PRMT5 is a major type II protein arginine methyltransferase that catalyzes symmetric dimethylation (SDMA) of histone and non-histone substrates [9,10,11] and regulates diverse cellular processes, including transcription [12], alternative splicing [13], signal transduction [14], and the DNA damage response [15]. PRMT5 is overexpressed in multiple cancer types, underscoring its therapeutic relevance [16,17].

First-generation PRMT5 inhibitors, including substrate-competitive and SAM-competitive compounds such as EPZ015666 [18] and JNJ-64619178 [19], exhibit limited selectivity between *MTAP*-WT and *MTAP*-null cancer cells because they effectively inhibit the SAM-bound form of PRMT5 that is also present in *MTAP*-WT cells. In contrast, second-generation MTA-cooperative PRMT5 inhibitors, such as BMS-986504/MRTX1719 (hereafter referred to as MRTX1719) [20,21,22], AMG 193 [23,24,25] and TNG908 [26,27], preferentially target the MTA-bound form of PRMT5 enriched in *MTAP*-null cancer cells while largely sparing *MTAP*-WT cells. This mechanism confers tumor-selective inhibition and helps overcome the limited therapeutic window associated with first-generation PRMT5 inhibitors. In phase I/Ib clinical trials, the tumor-selective PRMT5 inhibitors MRTX1719 and AMG 193 demonstrated objective response rates (ORRs) of 23% and 21.4%, respectively, with all reported responses being partial responses at well-tolerated dose levels [25,28]. However, these modest ORRs suggest that intrinsic or acquired resistance may limit the clinical efficacy of this therapeutic strategy.

Acquired resistance to first-generation PRMT5 inhibitors has been described in multiple preclinical models, including EPZ015666-resistant murine and human NSCLC cells [29,30] and PRT-382-resistant mantle cell lymphoma (MCL) cells [31]. Reported resistance mechanisms include upregulation of stathmin-2 (*STMN2*) or *PRMT5* mutations in EPZ015666-resistant cells, and activation of multiple signaling pathways, including mechanistic target of rapamycin (mTOR) signaling, in PRT-382-resistant MCL cells. Together, these findings highlight the need to define the molecular determinants of response to MTA-cooperative PRMT5 inhibitors and to identify rational therapeutic strategies that improve the depth and durability of clinical benefit.

In the present study, we developed in vitro models of acquired resistance to the MTA-cooperative PRMT5 inhibitor MRTX1719 in *MTAP*-null NSCLC cells. Using these models, we defined molecular features of the resistant state and identified candidate vulnerabilities arising during prolonged PRMT5 inhibition. Collectively, these findings provide preliminary insight into the molecular basis of acquired resistance and support further evaluation of targeting these vulnerabilities as a strategy to overcome resistance to MTA-cooperative PRMT5 inhibition in the clinic.

## 2. Materials and Methods

### 2.1. Cell Culture

Human NSCLC cell lines H1299 (#CRL-5803, RRID: CVCL_0060, sex: male), H1975 (#CRL-5908, RRID: CVCL_1511, sex: female), A549 (#CCL-185, RRID: CVCL_0023, sex: male), H838 (#CRL-5844, RRID: CVCL_1594, sex: male), H1437 (#CRL-5872, RRID: CVCL_1472, sex: male), and H2126 (#CCL-256, RRID: CVCL_1532, sex: male); the human SCLC cell line H2171 (#CRL-5929, RRID: CVCL_1536, sex: male); human colorectal cancer cell lines RKO (#CRL-2577, RRID: CVCL_0504, sex: unspecified) and DLD1 (#CCL-221, RRID: CVCL_0248, sex: male); the human melanoma cell line A375 (#CRL-1619, RRID: CVCL_0132, sex: female); and the murine melanoma cancer cell lines B16-F10 (#CRL-6475, RRID: CVCL_0159, sex: male) were obtained from the American Type Culture Collection (ATCC, Manassas, VA, USA). The murine fibrosarcoma cell line MCA205 (#SCC173, RRID: CVCL_VR90) and murine colon adenocarcinoma cell line MC38 (#SCC172, RRID: CVCL_B288, sex: female) were purchased from Sigma-Aldrich (St. Louis, MO, USA). All cell lines were maintained in the recommended media provided by the manufacturer at 37 °C in a humidified incubator with 5% CO_2_ and were confirmed to be free of mycoplasma contamination prior to use using the MycoAlert Mycoplasma Detection Kit (Lonza, Walkersville, MD, USA; #LT07-705).

### 2.2. Drug Treatment

PRMT5 inhibitors (EPZ015666 and MRTX1719), MTA and MEK inhibitors (trametinib and selumetinib) were dissolved in DMSO. EPZ015666 was purchased from Sigma-Aldrich (#SML1421) and MRTX1719 from ChemieTek (Indianapolis, IN, USA; #CT-MRTX1719). MTA was purchased from Sigma-Aldrich (#260585). Trametinib (#HY-10999) and selumetinib (#HY-50706) were purchased from MedChemExpress (Monmouth Junction, NJ, USA). Cells were treated with different concentrations of individual drugs or drug combinations for 5 or 6 days prior to Western blot analysis and cell viability assays. DMSO was used as the vehicle control.

### 2.3. Generation of MTAP-Isogenic Cell Lines

Human *MTAP*-knockout (*MTAP*-KO) cells were generated by infecting Cas9-expressing cells with lentiviruses produced from LentiGuide-Hygro-eGFP (Addgene, Watertown, MA, USA; #99375) or LentiGuide-BSD vectors carrying sgRNAs targeting the human *MTAP* gene. Following hygromycin or blasticidin selection, single-cell clones were isolated, expanded, and validated for loss of MTAP expression by Western blot analysis. Murine *MTAP*-KO cells were generated by transfection of sgRNA:Cas9 ribonucleoprotein (RNP) complexes purchased from Integrated DNA Technologies (IDT, Coralville, IA, USA) using Lipofectamine CRISPRMAX (Invitrogen, Thermo Fisher Scientific, Waltham, MA, USA; #CMAX00015). After transfection, single-cell clones were isolated, expanded, and validated for loss of MTAP expression by Western blot analysis. sgRNA sequences targeting human and mouse *MTAP* were designed using the GuidePro v2.1.0 tool [32]. The sgRNA sequences used were TCTGCCCGGGAGCTAAAACG for human *MTAP* and GGACAATAGTCACAATTGAG for mouse *MTAP*.

### 2.4. Generation of MRTX1719-Resistant Cell Lines

H838, A549 and H1437 cells were counted using a Cellometer K2 automated cell counter (Revvity, Lawrence, MA, USA) and seeded at 1 × 10^5^ cells, 1 × 10^5^ cells, 2 × 10^5^ cells per 10 cm dish, respectively. Cells were initially treated with MRTX1719 at initial concentrations of 0.02 μM for H838, 0.05 μM for A549, and 0.1 μM for H1437 cells. DMSO treatment was included for each cell line to control for potential solvent effects on drug response. Fresh drug-containing medium was replaced on Day 3 after treatment. On Day 7, cells were counted and reseeded in 10 cm dishes at their respective initial seeding densities in medium containing the same MRTX1719 concentration.

MRTX1719 was administered using a stepwise, cell line-specific, non-strict dose-escalation schedule. Each treatment stage was maintained for approximately 4 weeks. Dose escalation was guided by cell growth and viability; when substantial growth suppression was observed, the current concentration was maintained for an additional treatment stage before further escalation. The concentrations used across the six treatment stages were 0.02, 0.05, 0.1, 0.2, 0.5, and 1 μM for H838 cells; 0.05, 0.05, 0.1, 0.2, 0.5, and 1 μM for A549 cells; and 0.1, 0.2, 0.2, 0.4, 1, and 2 μM for H1437 cells. The resulting MRTX1719-resistant (MRTXR) cells were subsequently maintained in drug-free medium. Their sensitivity to MRTX1719 was periodically evaluated by cell viability assays relative to the corresponding DMSO-treated control (DMSO) cells.

### 2.5. Cell Viability Assay and Synergy Scoring

To determine the optimal seeding density, human and murine cell lines were initially seeded in 96-well plates at different densities without drug treatment and monitored for 6 days using Incucyte S3 (Sartorius, Ann Arbor, MI, USA) or Celigo (Revvity) confluence scanning. Based on the growth curves, cells were subsequently seeded in 96-well plates at the appropriate density in 100 µL complete medium and allowed to adhere for 14 h prior to treatment. PBS was added to the outer wells of each 96-well plate to minimize edge effects.

The following day, each drug was serially diluted (3.162-fold dilution) in DMSO. Each dilution was supplemented with additional DMSO to maintain equal DMSO concentrations across all treatments. Cells were then treated with 100 µL of the single-drug dilution series and monitored daily using Incucyte S3. Each treatment condition was performed in triplicate. After 6 days of treatment, cell viability was measured using the CellTiter-Glo (CTG) assay (Promega, Madison, WI, USA, #G7571) according to the manufacturer’s instructions. Luminescence signals were recorded using a GloMax Navigator microplate reader (Promega). Percent inhibition values were calculated by normalizing the relative luminescence unit (RLU) values from treated wells to the average RLU values of DMSO-treated control wells. Dose–response curves were generated by plotting log(inhibitor) versus response using a variable slope (four-parameter) model in GraphPad Prism 10.3.1 to determine IC50 values. For *MTAP*-isogenic cell pairs treated with EPZ015666 or MRTX1719, confluence measurements obtained from Incucyte S3 scanning were used to calculate percent inhibition and IC50 values using the same normalization and curve-fitting methods as the CTG assay.

For drug combination treatments, MRTX1719 was combined with trametinib or selumetinib at the indicated concentrations. The concentrations were selected based on the responses observed in single-drug treatments. Cell viability was determined using CTG assay, and synergy mean scores were calculated using the Bliss independence model with the SynergyFinder+ (v07.09.2024-R-3.10.3) tool [33]. Scores < −10 indicate likely antagonistic interactions, scores between −10 and 10 indicate likely additive effects, and scores > 10 indicate likely synergistic interactions between the two drugs.

For MRTX1719 treatment with or without MTA supplementation, paired DMSO-treated control and MRTXR cells were seeded in 100 μL of medium containing 10 μM MTA or no added MTA and incubated overnight. The 10 μM MTA pretreatment was selected as an exploratory proof-of-concept condition informed by LC–MS measurements described below showing extracellular MTA levels of up to approximately 200 ng per million cells in H838-DMSO cultures. This condition corresponded to 1 nmol (297.33 ng) of MTA per well and was not derived from a formal dose-ranging study. The following day, 100 μL of medium containing the indicated concentrations of MRTX1719 was added without removing the pretreatment medium. Cell viability was measured using CellTiter-Glo after 6 days of MRTX1719 treatment.

### 2.6. Western Blot

Cell pellets or adherent cells in culture plates were lysed using SDS lysis buffer (2% SDS, 10 mM Tris-HCl, 1 mM EDTA, pH 8.0), followed by heating at 98 °C for 10 min and vortexing every 2 min to ensure complete DNA shearing. Protein concentrations were determined using the Pierce BCA Protein Assay (Thermo Fisher Scientific, #23227) according to the manufacturer’s instructions. Equal amounts of protein were separated by SDS-PAGE and transferred onto nitrocellulose membranes using the Trans-Blot Turbo transfer system (Bio-Rad Laboratories, Hercules, CA, USA). Membranes were blocked in 5% milk in TBST and incubated with primary antibodies overnight at 4 °C on a rocking platform. The following day, membranes were incubated with HRP-conjugated secondary antibodies at room temperature for 1 h. Chemiluminescent signals were detected using an Amersham ImageQuant 800 imaging system (Cytiva, Marlborough, MA, USA).

The following antibodies were used at the indicated dilutions: arginine methylation marks (SDMA, MMA, and ADMA; 1:2500) [34]; PRMT5 (1:2000, Cell Signaling Technology, Danvers, MA, USA; #79998, RRID: AB_2799945); MTAP (1:1000, Cell Signaling Technology, #4158, RRID: AB_1904054); phospho-MEK1/2 (Ser217/221) (1:1000, Cell Signaling Technology, #9154, RRID: AB_2138017); MEK1/2 (1:2000, Cell Signaling Technology, #9122, RRID: AB_823567); phospho-p44/42 MAPK (ERK1/2) (1:1000, Cell Signaling Technology, #9101, RRID: AB_331646); p44/42 MAPK (ERK1/2) (1:2000, Cell Signaling Technology, #4695, RRID: AB_390779); α-tubulin (1:5000, Developmental Studies Hybridoma Bank, Iowa City, IA, USA; #12G10); β-actin (1:5000, Sigma-Aldrich, #A1978, RRID: AB_476692); mouse IgG HRP-linked secondary antibody (1:10,000, Cytiva, #NA931, RRID: AB_772210); and rabbit IgG HRP-linked secondary antibody (1:10,000, Cytiva, #NA934, RRID: AB_772206).

### 2.7. Measurement of MTA Levels by LC-MS

3 × 10^5^ H838 and 4.5 × 10^5^ H1437 parental (DMSO) and MRTX1719-resistant (MRTXR) paired cells were seeded into individual wells of 6-well plates containing 1 mL of complete medium. Each cell line was seeded in biological triplicate. After 24 h, cell pellets and culture supernatants were collected for MTA quantification, and cell numbers were counted for normalization. MTA levels were measured using an AB SCIEX QTRAP 5500 mass spectrometer (SCIEX, Marlborough, MA, USA) coupled to a Shimadzu UPLC system (Shimadzu Scientific Instruments, Columbia, MD, USA). Sample preparation and LC–MS/MS analysis were performed as previously described using MTA mass transition *m*/*z* 298.2 → 136.1 in positive ion mode [35]. Intracellular (cell pellet) and extracellular (culture supernatant) MTA concentrations were quantified by comparison with a standard curve generated from external reference standards prepared in the mobile phase and normalized to the corresponding cell numbers.

### 2.8. High-Throughput Drug Screening

A high-throughput drug screen was performed using a compound library consisting of 619 compounds, including 59 SGC epigenetic compounds, 380 TargetMol epigenetic inhibitors and 180 FDA-approved oncology drugs. Drug libraries were diluted in DMSO to generate 10 mM stock solutions and arrayed onto Echo-certified low dead volume (LDV) plates. The Combinatorial Drug Discovery Program (CDDP) at Texas A&M University maintains this compound library and performed the drug screening. For the screening assays, approximately 300 cells from H838 and H1437 DMSO and MRTXR cell lines were seeded into Greiner black 384-well plates in growth medium using a Multidrop Combi liquid dispenser and cultured at 37 °C in a humidified incubator with 5% CO_2_ overnight. The next day, cells were treated with compounds by transferring materials from the LDV source plates into assay plates using the Labcyte Echo 550 platform. Cells were then exposed to the drug library at three concentrations (0.1 μM, 1 μM, and 10 μM) for 6 days. An 8-dose series of MRTX1719 was included as a positive control, and an 8-dose series of anisomycin was included as a nonselective control for drug response. At the endpoint, cells were washed, fixed, and stained with DAPI. Assay plates were imaged using a 4× objective on an ImageXpress microconfocal system, which captures the entire well area in a single field of view. Automated image analysis was performed using the advanced imaging collection in Biovia Pipeline Pilot to perform background correction and segment nuclei based on the DAPI signal. Drug screening was performed twice independently as biological replicates and drug responses between DMSO and MRTXR cells were evaluated using two-way ANOVA analysis based on the Hafner growth rate index (GRI) [36] derived from cell counts to determine whether dose–response curves exhibited statistically significant drug effects. Significant drug candidates were identified based on response differences (MRTXR vs. DMSO) greater than 0.25 or less than −0.25 with an adjusted *p*-value < 0.05.

### 2.9. RNA Sequencing and Data Analysis

RNA was isolated from H838 and H1437 DMSO and MRTXR cells in triplicate using the RNeasy Mini Kit (QIAGEN, Germantown, MD, USA; #74104) according to the manufacturer’s instructions. In-column DNase treatment was performed during RNA isolation. cDNA library preparation and RNA sequencing were performed by Signios Bio (Foster City, CA, USA) using the Illumina TruSeq Stranded mRNA Library Prep Kit (Illumina, San Diego, CA, USA), and sequencing was conducted on a NovaSeq X Plus platform (Illumina, San Diego, CA, USA). Each sample generated approximately 80 million paired-end reads of 150 bp in length. FASTQ files were mapped to the human reference genome (hg38) using TopHat (v2.0.10) and Bowtie (v2.1.0) with default parameter settings. Differentially expressed genes (DEGs) were identified by comparing MRTXR cells to DMSO cells using DESeq2 with thresholds of |log_2_ (Fold Change), FC| > 1 and a false discovery rate (FDR) < 0.05. The significance of the overlap between the H838 and H1437 differentially expressed gene sets was assessed separately for upregulated and downregulated genes using one-sided Fisher’s exact tests. The union of detected protein-coding genes across the two cell-line datasets was used as the background gene universe (*N* = 15,339). KEGG pathway enrichment analysis was performed separately for upregulated and downregulated genes using Metascape v3.5 with default settings [37].

### 2.10. Statistical Analysis

Data are presented as mean ± SD. Statistical analyses were performed using GraphPad Prism 10.3.1. Statistical significance of changes in MTA levels was determined using an unpaired two-tailed t test. Statistical significance is indicated as follows: n.s., not significant; * *p* < 0.05; ** *p* < 0.01; *** *p* < 0.001.

## 3. Results

### 3.1. MRTX1719 Selectively Inhibits MTAP-Null Cells with Variable Responses

**Figure 1:**
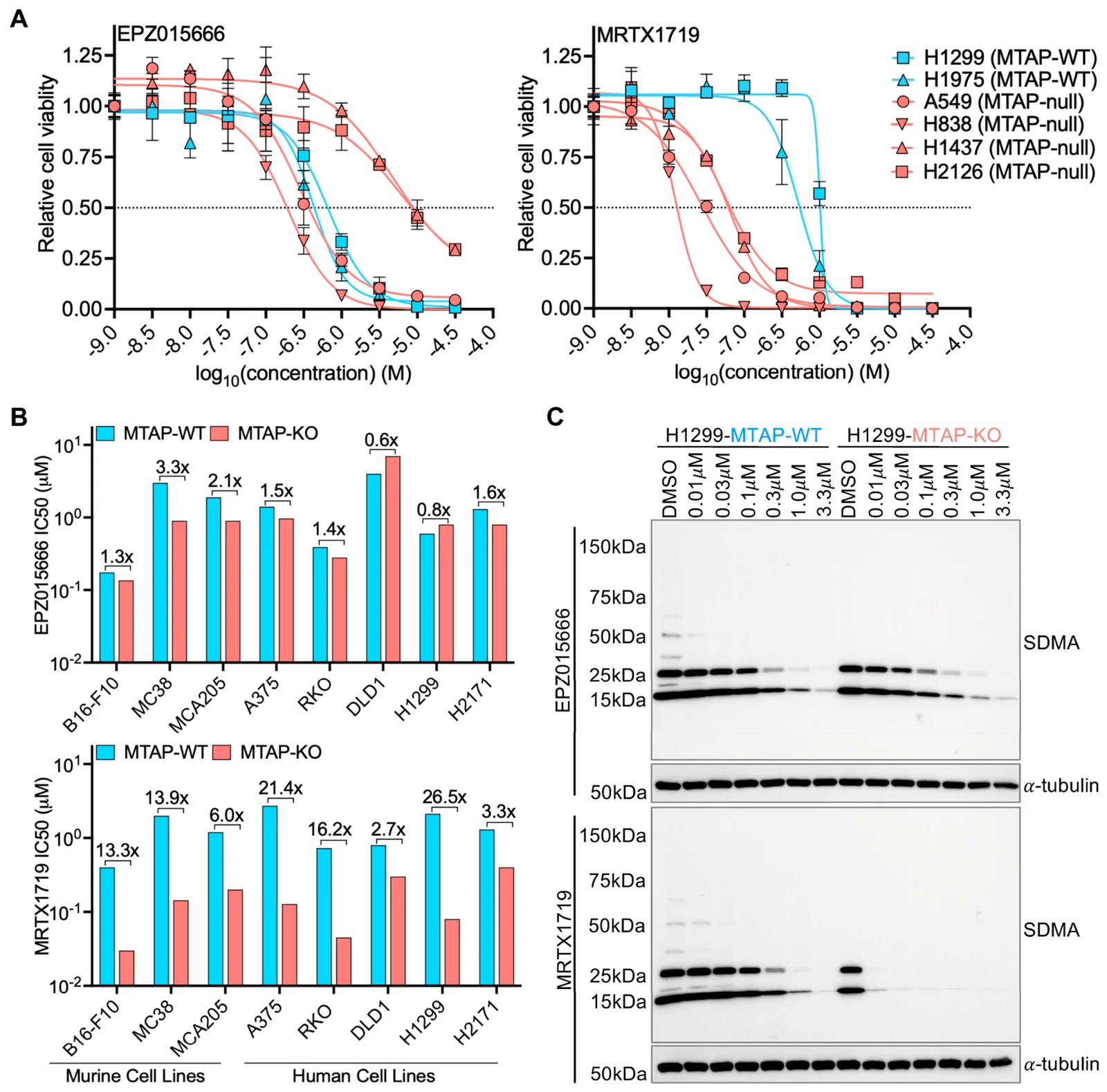
Selective but heterogeneous inhibition of *MTAP*-null cells by MRTX1719. (**A**) Dose–response curves of *MTAP*-WT and *MTAP*-null NSCLC cell lines treated with EPZ015666 or MRTX1719 for 6 days. Data are presented as mean ± SD. (**B**) Comparison of IC50 values for EPZ015666 (top) and MRTX1719 (bottom) in *MTAP*-isogenic murine and human cell lines. Fold differences in IC50 between *MTAP*-WT and *MTAP*-null cells are indicated. (**C**) Immunoblot analysis of SDMA levels in the H1299 *MTAP*-isogenic cell pair following treatment with increasing concentrations of EPZ015666 or MRTX1719.

MRTX1719, an MTA-cooperative PRMT5 inhibitor, has been reported to selectively target *MTAP*-null cancer models and patient tumors [21]. Consistent with this, MRTX1719 preferentially reduced viability in a panel of *MTAP*-null NSCLC cell lines after 6 days of treatment, whereas the substrate-competitive PRMT5 inhibitor EPZ015666 showed no *MTAP*-selective activity (Figure 1A). MTAP status in these NSCLC models was confirmed by immunoblotting (Figure S1A).

To directly assess the contribution of MTAP loss to drug selectivity and sensitivity, we generated eight murine and human *MTAP*-isogenic cell line pairs spanning multiple tumor types using CRISPR-mediated *MTAP* knockout and confirmed MTAP loss by immunoblotting (Figure S1B, Supplementary Table S1). Following 6 days of treatment, MRTX1719 generally produced a greater IC50 shift (2.7-fold to 26.5-fold) between *MTAP*-WT and *MTAP*-KO cells than EPZ015666 (0.6-fold to 3.3-fold), although the magnitude of this effect varied substantially by model (Figure 1B). Notably, human DLD1 and H2171 *MTAP*-KO cells showed relatively limited sensitivity to MRTX1719 compared with other *MTAP*-KO models, suggesting that MTAP loss alone does not uniformly predict sensitivity in *MTAP*-null contexts.

Consistent with these growth effects, MRTX1719, but not EPZ015666, selectively reduced PRMT5 activity, as reflected by decreased SDMA levels, in H1299 and A375 *MTAP*-KO cells (Figure 1C and Figure S1C). By contrast, *MTAP*-WT cells were largely unaffected by MRTX1719 at concentrations below 1 μM. Together, these findings establish that MRTX1719 preferentially inhibits cell viability and PRMT5 activity in *MTAP*-null cells, although sensitivity varies across models.

### 3.2. Prolonged MRTX1719 Treatment Induces Acquired Resistance in Sensitive NSCLC Cells

**Figure 2:**
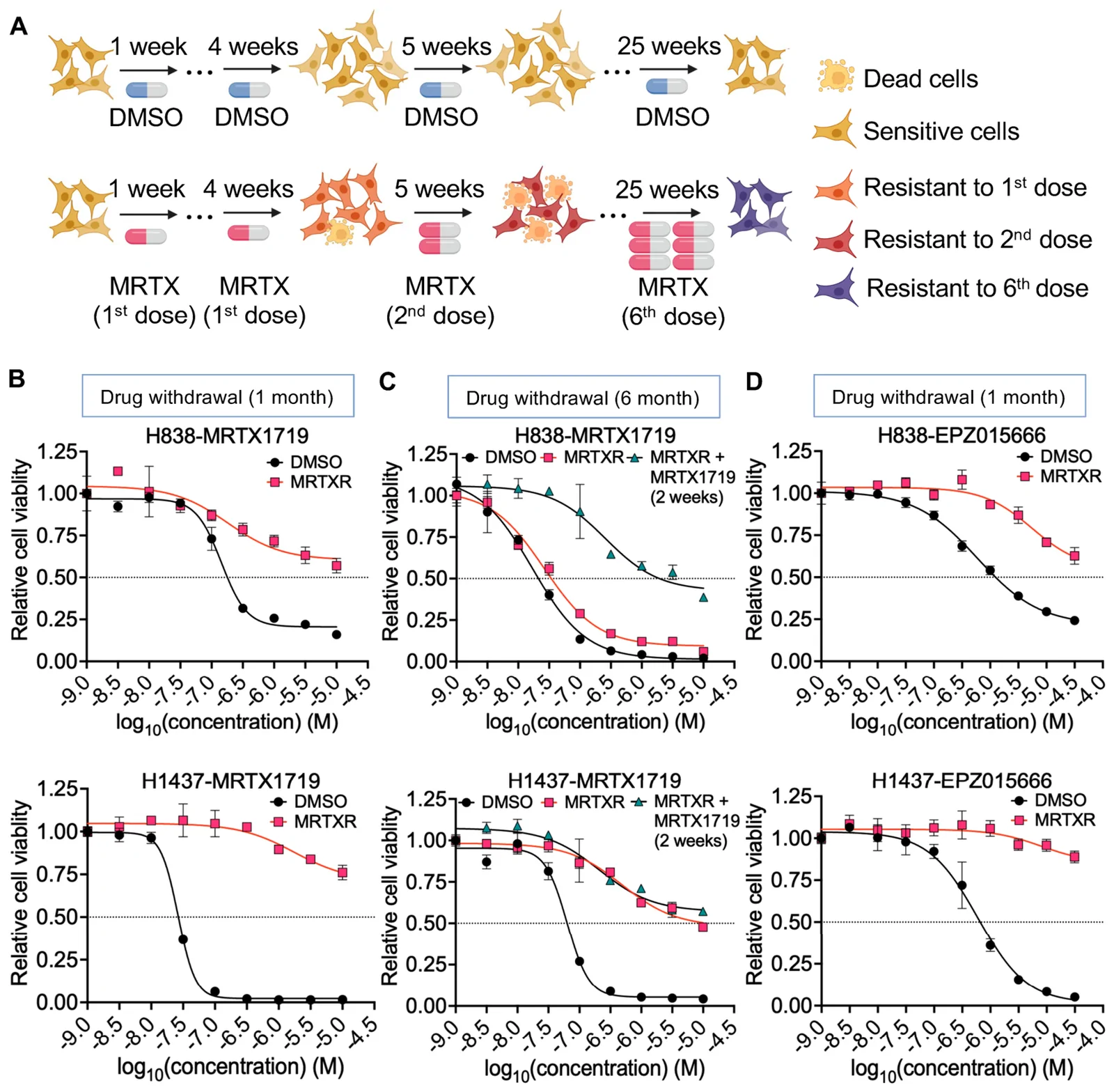
Acquired resistance to MRTX1719 in sensitive NSCLC cells in vitro. (**A**) Schematic illustrating the stepwise, cell line-specific dose-escalation strategy used to generate MRTX1719-resistant cells. MRTX1719 concentrations were adjusted at approximately 4-week intervals over a 25-week treatment period. (**B**) MRTX1719 dose–response assays in paired DMSO-treated control (DMSO) and MRTX1719-resistant (MRTXR) cells after 1 month of drug withdrawal following prolonged MRTX1719 treatment. (**C**) MRTX1719 dose–response assays in paired DMSO and MRTXR cells maintained in drug-free medium for approximately 6 cumulative months, and MRTXR cells rechallenged with MRTX1719 for 2 weeks after the drug-withdrawal period. (**D**) EPZ015666 dose–response assays in paired DMSO and MRTXR cells after 1 month of drug withdrawal. Data are presented as mean ± SD.

To investigate acquired MRTX1719 resistance, we generated resistant cells from the MRTX1719-sensitive NSCLC cell lines A549, H838, and H1437 using prolonged, stepwise, cell line-specific dose escalation strategy [38,39]. Starting concentrations were selected based on short-term dose–response profiles and were adjusted at approximately 4-week intervals according to cell growth and viability. Cells were initially treated with 0.02 μM MRTX1719 for H838, 0.05 μM for A549, and 0.1 μM for H1437. After 25 weeks, the final concentrations reached 1 μM in H838 and A549 cells and 2 μM in H1437 cells (Figure 2A and Figure S2A). Parallel long-term DMSO treatment served as a control for prolonged solvent exposure.

MRTX1719-treated cells became enlarged and exhibited markedly slower proliferation than DMSO-treated controls during resistance generation (Figure S2A,B). After long-term treatment, cells were cultured in drug-free medium for 1 month to allow growth recovery and were then tested for MRTX1719 sensitivity. Compared with DMSO-treated controls (DMSO), MRTX1719-treated cells (MRTXR) showed clear acquired resistance in H838 and H1437 cells (Figure 2B), whereas A549 cells exhibited only a modest shift in sensitivity (Figure S2C). Because of its relatively narrow resistance window, the A549-MRTXR model was not investigated further.

To assess the stability of the acquired resistance phenotype, H838-MRTXR and H1437-MRTXR cells were maintained under drug-free conditions. After approximately 6 cumulative months of proliferative culture in the absence of MRTX1719, H838-MRTXR cells had lost most of their resistance, whereas H1437-MRTXR cells retained resistance, indicating reversible and stable resistant states, respectively (Figure 2C). Following 2 weeks of MRTX1719 re-challenge, H838-MRTXR cells reacquired resistance, whereas H1437-MRTXR cells maintained a similar degree of resistance (Figure 2C). These results demonstrate that prolonged MRTX1719 treatment induces acquired resistance in sensitive NSCLC cells and that the resulting resistant states differ in their stability following drug withdrawal.

Meanwhile, to determine whether the acquired-resistant cells were also less sensitive to a mechanistically distinct PRMT5 inhibitor, we evaluated their responses to EPZ015666. Both H838-MRTXR and H1437-MRTXR cells showed reduced sensitivity to EPZ015666 relative to their paired DMSO-treated control cells (Figure 2D), demonstrating cross-resistance to an MTA-independent mode of PRMT5 inhibition. Thus, the acquired resistant phenotype was not restricted to reduced sensitivity to MRTX1719 alone.

### 3.3. MRTX1719 Resistance Is Not Fully Explained by Altered PRMT5 Activity or Changes in MTA Levels

**Figure 3:**
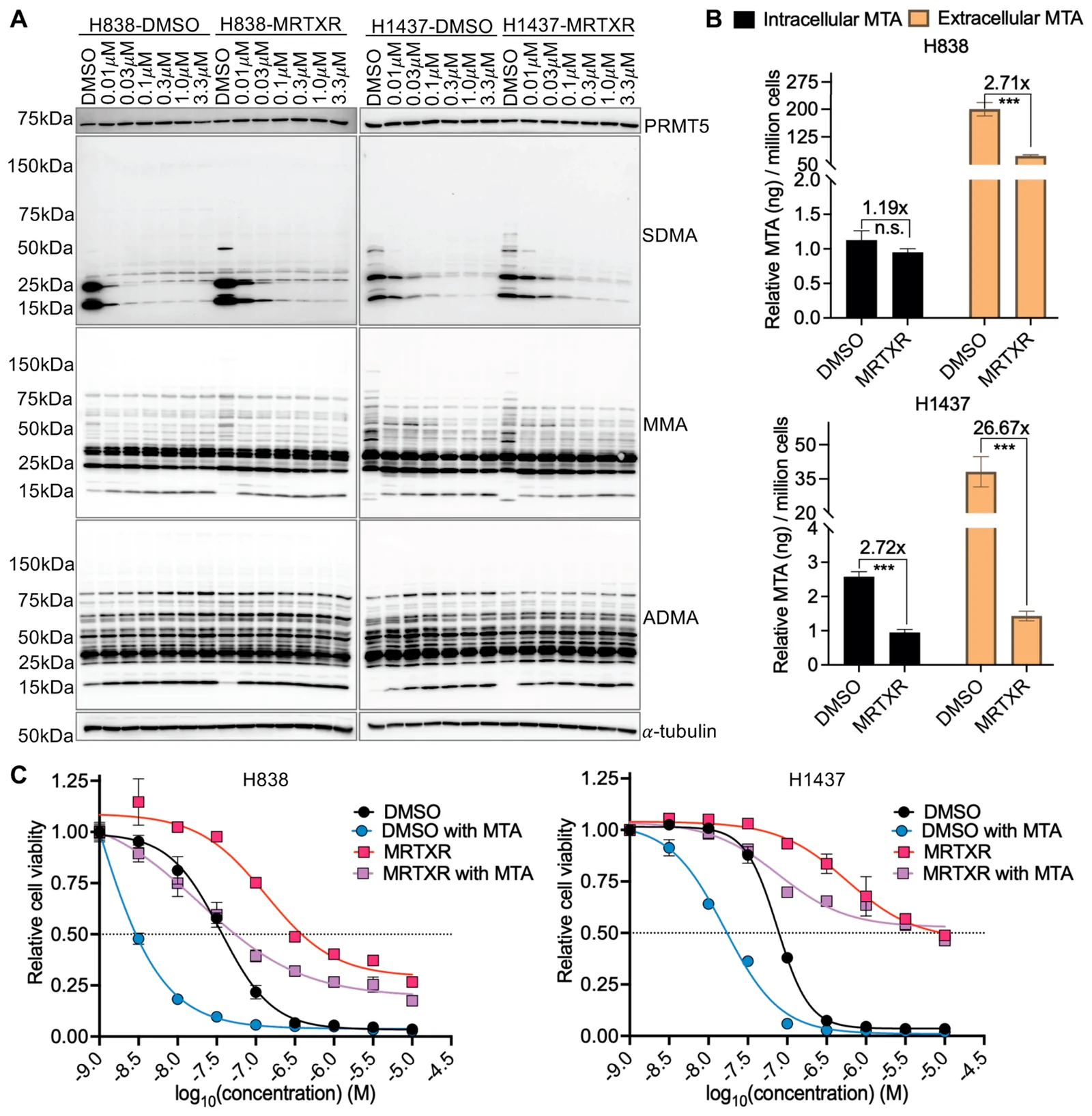
MRTX1719 resistance not fully explained by altered PRMT5 activity or reduced MTA levels. (**A**) Immunoblot analysis of PRMT5 and arginine methylation marks (SDMA, MMA, and ADMA) in DMSO and MRTXR cells treated with increasing concentrations of MRTX1719. (**B**) Quantification of intracellular and extracellular MTA levels in DMSO and MRTXR cells. (**C**) MRTX1719 dose–response assays in DMSO and MRTXR cells with or without MTA supplementation. Data are presented as mean ± SD. n.s., not significant; *** *p* < 0.001.

Having established MRTX1719-resistant models, we first examined whether acquired resistance was associated with altered PRMT5 activity. Paired DMSO and MRTXR cells were treated with increasing doses of MRTX1719 for 6 days. The dose-dependent suppression of SDMA was broadly similar between sensitive and resistant cells, with a slight delay in SDMA loss in resistant cells at concentrations below 0.1 μM. Basal SDMA levels and PRMT5 expression were also largely unchanged in resistant cells (Figure 3A). Several bands were more intense in resistant cells at baseline, but this pattern was lost after extended culture in drug-free medium, suggesting that it was transient. Consistent with these findings, time-course analysis following treatment with 0.1 μM MRTX1719 showed similar temporal reductions in SDMA levels in paired DMSO and MRTXR cells (Figure S3A). EPZ015666 treatment also produced broadly comparable dose-dependent suppression of SDMA in paired DMSO and MRTXR cells (Figure S3B), despite the reduced sensitivity of both resistant models to this mechanistically distinct PRMT5 inhibitor (Figure 2D).

Given the reported functional overlap between PRMT5 and PRMT1 [40,41,42], we also examined whether resistance was associated with compensatory changes in other PRMT activities. MMA and ADMA levels showed no obvious differences at baseline or after MRTX1719 treatment (Figure 3A), indicating that no major global compensatory changes in other PRMT activities were evident. In addition, Sanger sequencing of the *PRMT5* coding region identified no acquired coding mutations in either resistant model. Together, these findings suggest that resistance is not readily explained by overt changes in PRMT5 expression or catalytic suppression, global compensatory changes in other PRMT activities, or *PRMT5* coding mutations.

We next examined whether acquired resistance was associated with altered MTA levels. Intracellular and extracellular MTA levels were quantified by LC–MS in DMSO and MRTXR cell pellets and supernatants. Intracellular MTA was significantly reduced in H1437-MRTXR cells and showed a nonsignificant downward trend in H838-MRTXR cells, whereas extracellular MTA was significantly reduced in both resistant lines, with a more pronounced decrease in H1437-MRTXR (Figure 3B). To assess whether MTA supplementation modulated MRTX1719 response, paired DMSO and MRTXR cells were pretreated overnight with 10 μM MTA before MRTX1719 exposure and evaluated using a 6-day viability assay. MTA supplementation enhanced the growth-inhibitory effect of MRTX1719 in the DMSO control cells, consistent with the MTA-cooperative mechanism of MRTX1719. It also partially increased MRTX1719 sensitivity in the resistant cells but did not fully reverse the resistant phenotype (Figure 3C). Together with the observed cross-resistance to EPZ015666, these findings indicate that reduced MTA levels may contribute to reduced MRTX1719 sensitivity but are insufficient to fully account for the acquired resistant phenotype.

### 3.4. Acquired MRTX1719 Resistance Confers Collateral Sensitivity to MEK Inhibitors

Because altered PRMT5 activity and changes in MTA levels did not fully account for acquired resistance, we performed a high-throughput drug screen to identify therapeutic vulnerabilities associated with resistance state. The screening library comprised 619 compounds, including SGC epigenetic compounds, TargetMol epigenetic inhibitors, and FDA-approved oncology drugs (Figure S4A). Screen performance was supported by the expected responses to the positive control MRTX1719 and the nonselective control anisomycin (Figure S4B).

**Figure 4:**
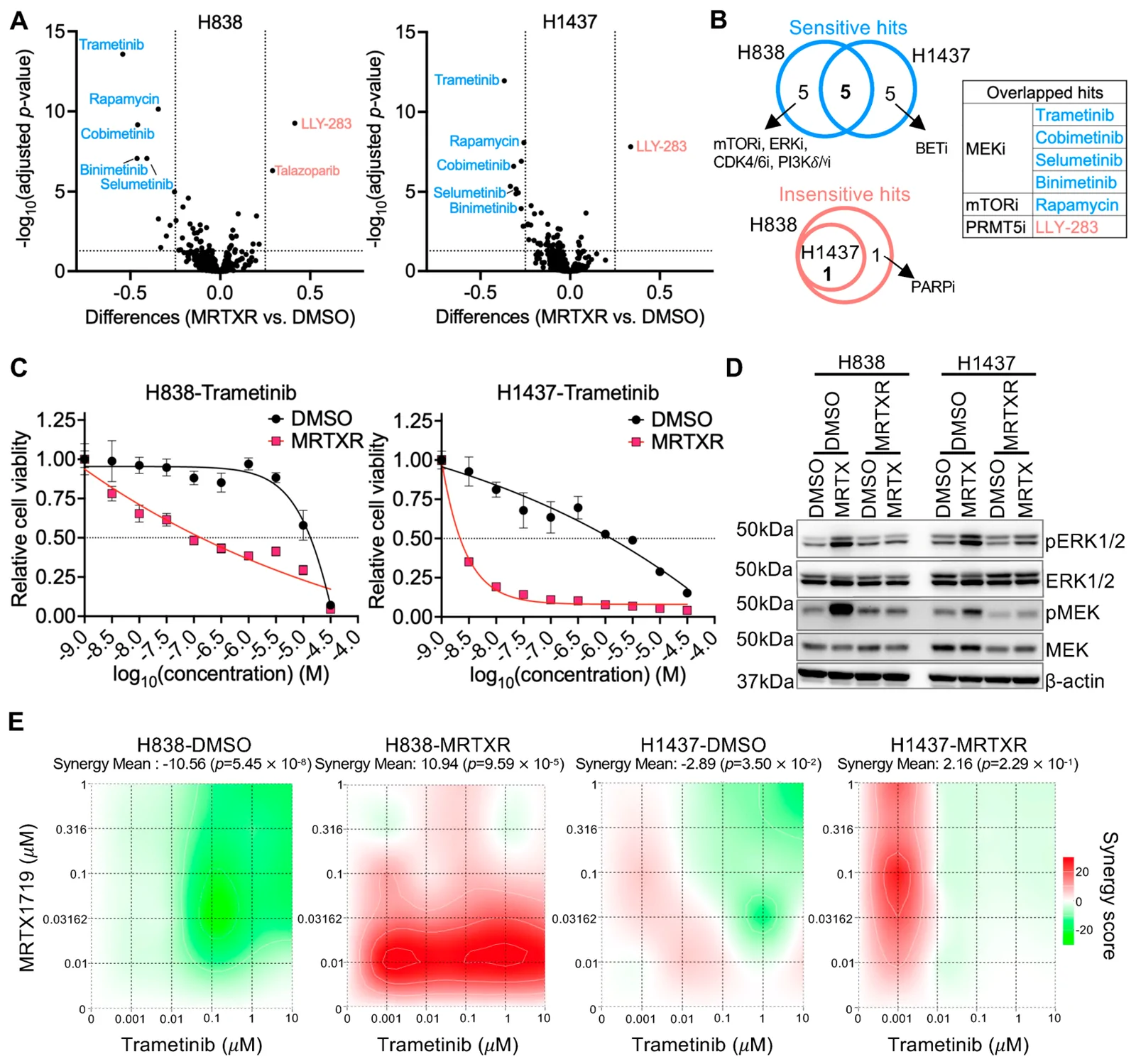
Collateral sensitivity to MEK inhibitors in MRTX1719-resistant NSCLC cells in vitro. (**A**) Volcano plots illustrating differential drug sensitivity between MRTXR and DMSO cells from the high-throughput drug screen. Significant hits were defined as viability differences (MRTXR vs. DMSO) >0.25 or <−0.25 with an adjusted *p*-value < 0.05. Blue dots indicate drugs to which MRTXR cells are more sensitive than DMSO cells, whereas red dots indicate drugs to which DMSO cells are more sensitive. (**B**) Venn diagram (left) and summary table (right) showing overlapping significant hits identified in both cell lines. (**C**) Dose–response curves of DMSO and MRTXR cells treated with the MEK inhibitor trametinib. Data are presented as mean ± SD. (**D**) Immunoblot analysis of total and phosphorylated ERK and MEK in DMSO and MRTXR cells with or without MRTX1719 treatment for 3 days. (**E**) Synergy analysis of MRTX1719 and trametinib in DMSO and MRTXR cells. Synergy mean scores were calculated using the Bliss model with the SynergyFinder+ tool.

Differential drug response analysis identified compounds to which H838- and H1437-MRTXR cells were either more or less sensitive than their corresponding controls (Figure 4A, Supplementary Tables S2 and S3). Five significantly sensitizing hits were shared between the two resistant lines, including four MEK inhibitors: trametinib, cobimetinib, selumetinib and binimetinib, and the mTOR inhibitor rapamycin. Additional mTOR, ERK, CDK4/6, and PI3Kδ/γ inhibitor hits were identified in H838-MRTXR cells, whereas multiple BET inhibitor hits were identified in H1437-MRTXR cells, indicating a broader, partly cell-line-specific sensitizing profile. In contrast, the SAM-competitive PRMT5 inhibitor LLY-283 was a shared reduced-sensitivity hit, further supporting cross-resistance to mechanistically distinct PRMT5 inhibitors (Figure 4B).

Because MEK inhibitors were the only drug class represented by multiple shared compounds across both resistant models and constituted the strongest cross-model, class-level signal in the screen, we prioritized MEK inhibition for validation. Dose–response assays confirmed that H838- and H1437-MRTXR cells were more sensitive than their paired controls to trametinib (Figure 4C) and selumetinib (Figure S4C), consistent with the screening results. These findings identify enhanced sensitivity to MEK inhibition as a reproducible collateral vulnerability associated with acquired MRTX1719 resistance.

We next examined MAPK signaling by assessing MEK and ERK phosphorylation. Basal MEK and ERK phosphorylation were not obviously increased in resistant cells relative to sensitive controls. However, after 3 days of MRTX1719 treatment, MEK and ERK phosphorylation increased in H838 and H1437 DMSO cells, whereas this response was blunted in MRTXR cells (Figure 4D). These findings indicate that short-term MRTX1719 treatment induces activation of MAPK/ERK signaling in sensitive cells, whereas this inducible response is altered following resistance acquisition.

We next evaluated combination treatment with MRTX1719 and the MEK inhibitors trametinib or selumetinib in paired DMSO and MRTXR cells. In the DMSO cells, mean synergy scores indicated additive or antagonistic interactions rather than synergy. In contrast, both combinations were synergistic in H838-MRTXR cells, with mean synergy scores exceeding 10. In H1437-MRTXR cells, the mean synergy scores remained within the additive range, although localized positive interactions were observed at selected low-dose combinations (Figure 4E and Figure S4D). Thus, the combination effects were model and inhibitor dependent and did not support a consistently effective upfront combination strategy across the tested models.

### 3.5. MAPK-Related Transcriptional Programs Are Enriched in MRTX1719-Resistant Cells

**Figure 5:**
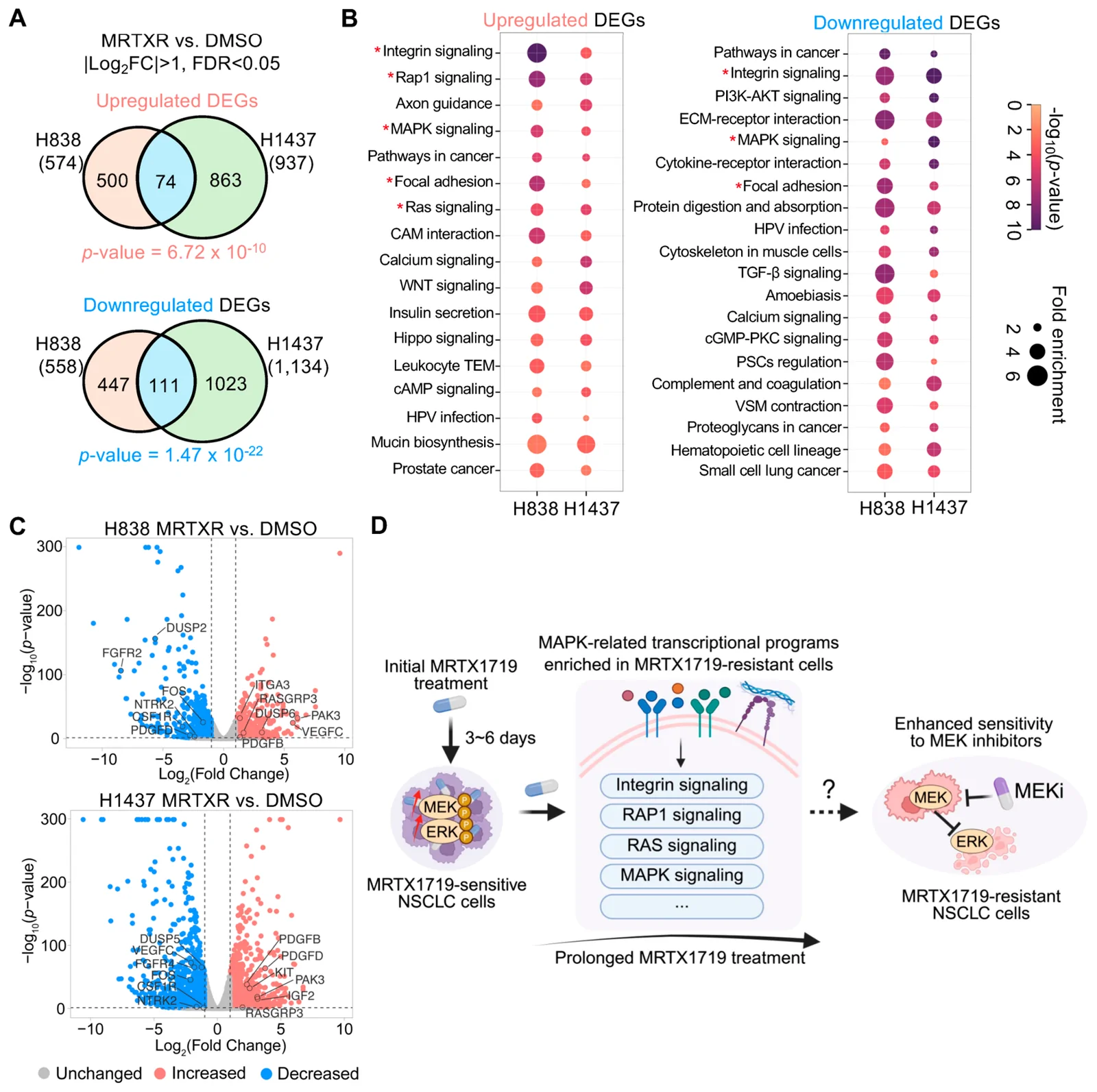
Enriched MAPK-related transcriptional programs in MRTX1719-resistant cells. (**A**) Venn diagram showing the number of overlapping differentially expressed genes (DEGs) identified by RNA-seq analysis in both cell lines. DEGs were defined as coding genes with |log_2_ (fold change, FC)| > 1 between MRTXR and DMSO cells with FDR < 0.05. Fisher’s exact test was used to assess the significance of the overlap between the two cell models for upregulated genes (*p*-value = 6.72 × 10^−10^) and downregulated genes (*p*-value = 1.47 × 10^−22^). (**B**) KEGG pathway enrichment analysis of upregulated (left) and downregulated (right) DEGs identified in each cell line. The top 20 enriched pathways are shown for downregulated DEGs. MAPK-related pathways are marked with asterisks. (**C**) Representative DEGs in MAPK-related transcriptional programs shown in volcano plots. (**D**) Proposed model illustrating that MRTX1719 initially activates MAPK/ERK signaling, and continued long-term treatment is associated with the emergence of a resistant state characterized by enrichment of MAPK-related transcriptional programs and enhanced sensitivity to MEK inhibition. The mechanistic relationship between these features remains unresolved.

To characterize global transcriptional changes associated with acquired MRTX1719 resistance, we performed RNA sequencing in DMSO- and MRTXR-paired cells. Using |log_2_ fold change| > 1 and FDR < 0.05 to define significantly differentially expressed coding genes in MRTXR relative to DMSO cells, we identified 574 upregulated genes in H838 and 937 in H1437, with 74 genes shared between the two models. Similarly, 558 downregulated genes in H838 and 1134 in H1437 were identified, with an overlap of 111 genes (Figure 5A, Supplementary Tables S4 and S5). Fisher’s exact test indicated that the overlaps between the two cell models were significant for both upregulated (*p*-value = 6.72 × 10^−10^) and downregulated genes (*p*-value = 1.47 × 10^−22^).

We next performed KEGG pathway enrichment analysis using Metascape [37] on the upregulated and downregulated gene sets from each cell line. MAPK-related pathways, including MAPK signaling itself as well as upstream or associated pathways such as RAS, RAP1, integrin, and extracellular adhesion signaling, were significantly enriched in both resistant models (Figure 5B, Supplementary Table S6). Representative differentially expressed genes in these pathways were shown in the volcano plots (Figure 5C). Notably, both positive and negative regulators of MAPK-related pathways were differentially expressed in resistant cells, indicating transcriptional remodeling rather than uniform pathway upregulation.

Together, these data identify enrichment of MAPK-related transcriptional programs as a shared feature of MRTX1719-resistant cells and support an association between the resistant state and collateral sensitivity to MEK inhibition. Based on these observations, we propose a working model in which short-term MRTX1719 treatment increases MEK-ERK phosphorylation in sensitive cells, whereas prolonged treatment is associated with the emergence of a resistant state characterized by MAPK-related transcriptional remodeling and enhanced sensitivity to MEK inhibition. The mechanistic relationship between these features remains unresolved (Figure 5D).

## 4. Discussion

In this study, we generated multiple *MTAP*-isogenic cell models to evaluate the contribution of MTAP loss to MRTX1719 efficacy. Although MRTX1719 showed preferential activity in *MTAP*-KO cells relative to *MTAP*-WT counterparts, responses among *MTAP*-KO models were variable. These findings demonstrate that MTAP loss alone is insufficient to predict MRTX1719 sensitivity, consistent with the modest ORRs observed clinically [43]. Future studies should therefore focus on identifying predictive biomarkers and rational combination strategies to improve the efficacy of MRTX1719 more broadly. In this context, several combinations of MRTX1719 with chemotherapy, targeted therapy and immunotherapy are currently under clinical evaluation [43].

To investigate acquired resistance to MRTX1719 and identify associated vulnerabilities, we established resistant cell models through prolonged drug exposure. The resulting models displayed distinct resistance phenotypes. A549-MRTXR cells exhibited a narrower resistance window than H838-MRTXR and H1437-MRTXR cells and were therefore not investigated further. H838-MRTXR cells showed a reversible resistant state following drug withdrawal, whereas H1437-MRTXR cells retained a stable resistant phenotype. The determinants of these distinct resistance states remain unresolved. The reversible H838 phenotype may reflect a drug-dependent adaptive state, whereas the stable H1437 phenotype may involve more durable molecular alterations, as reported for resistance to other targeted therapies [44,45,46,47,48,49,50]. Because genomic analysis in this study was limited to the PRMT5 coding region, future studies incorporating whole-genome sequencing, transcriptomic analysis, and epigenomic profiling will be required to define the genetic and non-genetic determinants of these resistance states. Validation in additional *MTAP*-null models and cancer lineages will also be necessary to determine the generalizability of these findings.

Because MRTX1719 is an MTA-cooperative PRMT5 inhibitor, reduced intracellular MTA represents a plausible contributor to diminished drug response. Intracellular MTA was significantly reduced in H1437-MRTXR cells, whereas H838-MRTXR cells showed only a nonsignificant downward trend, indicating that reduced intracellular MTA may not be a shared feature of acquired resistance in the two models. Extracellular MTA was significantly reduced in both resistant models, although extracellular levels do not directly reflect the intracellular MTA pool available for MRTX1719-dependent PRMT5 engagement. Exogenous MTA supplementation increased MRTX1719 sensitivity in the resistant cells but did not fully reverse resistance. Thus, MTA availability can modulate the response to MRTX1719, but reduced MTA alone is insufficient to account fully for the acquired resistant phenotype.

Both resistant models also showed reduced sensitivity to EPZ015666, a mechanistically distinct PRMT5 inhibitor whose activity does not require the MTA-bound form of PRMT5. Reduced sensitivity to another MTA-independent PRMT5 inhibitor, LLY-283, was also identified in the drug screen. Moreover, MRTX1719 and EPZ015666 produced broadly comparable SDMA suppression in paired DMSO and MRTXR cells, indicating that pharmacological suppression of PRMT5 catalytic activity was retained. Together, these findings suggest that reduced MTA may influence MRTX1719 sensitivity, but the resistant phenotype also involves adaptations extending beyond impaired MTA-dependent drug engagement. The basis of the significantly reduced intracellular MTA level observed in H1437-MRTXR cells also warrants further investigation.

A key finding of this study was that acquired MRTX1719 resistance was accompanied by collateral sensitivity to multiple MEK inhibitors. RNA-sequencing analysis further revealed enrichment of MAPK-related transcriptional programs in both resistant models. However, whether these transcriptional changes contribute to resistance or enhanced MEK inhibitor sensitivity remains unclear. At the signaling level, basal pMEK and pERK levels were not obviously elevated, and short-term MRTX1719-induced MEK-ERK phosphorylation was attenuated in resistant cells relative to their paired control cells. These observations argue against sustained MAPK pathway hyperactivation in the resistant state. Nevertheless, pathway activity and functional pathway dependence are not necessarily equivalent. The enhanced sensitivity of both resistant models to multiple MEK inhibitors is consistent with, but does not establish, greater reliance on MEK-ERK signaling for growth or viability. One possibility is that resistant cells become more vulnerable to disruption of residual MEK-ERK activity despite having a reduced capacity for further pathway induction. This hypothesis remains speculative, and genetic perturbation or rescue experiments will be required to determine whether increased MEK-ERK dependence underlies the observed collateral sensitivity. Thus, the mechanistic relationships among altered MEK-ERK phosphorylation dynamics, MAPK-related transcriptional remodeling, and enhanced MEK inhibitor sensitivity in resistant cells remains unresolved.

Previous studies have reported synergy between MRTX1719 and trametinib in *KRAS*-mutant NSCLC parental models and between MRTX1719 and KRAS inhibition in *KRAS*-mutant pancreatic ductal adenocarcinoma (PDAC) models [51,52]. In contrast, MRTX1719 combined with trametinib or selumetinib produced additive or antagonistic interactions in the DMSO-treated control cells examined here. Both combinations were synergistic in H838-MRTXR cells, whereas mean synergy scores in H1437-MRTXR cells remained within the additive range despite localized positive interactions at selected low-dose combinations. These differences may reflect variation in oncogenic background, signaling context, and resistance state. Unlike the previously studied *KRAS*-mutant models, H838 and H1437 cells are *KRAS*-wildtype. Thus, our findings do not exclude the potential benefit of combination treatment in other molecular contexts but do not support a consistently effective combination strategy across the models tested.

Despite these variable combination effects, both H838-MRTXR and H1437-MRTXR cells were more sensitive to MEK inhibitor monotherapy than their paired DMSO cells. This shared collateral sensitivity raises the possibility that MEK inhibition may be more relevant as a treatment switch following the emergence of MRTX1719 resistance than as an upfront combination in the models tested. This proposed sequential strategy will require further validation in in vivo treatment-switching studies and additional *MTAP*-null cancer models.

## 5. Conclusions

In summary, our findings demonstrate that response to the MTA-cooperative inhibitor MRTX1719 is selective but heterogeneous. Using in vitro-derived MRTX1719-resistant models, we found that acquired resistance was not fully explained by altered PRMT5 activity or changes in MTA levels and was ac-companied by cross-resistance to mechanistically distinct PRMT5 inhibitors. Acquired resistance was also associated with collateral sensitivity to MEK inhibition and enrichment of MAPK-related transcriptional programs.

## Data Availability

The data generated in this study are available upon request from the corresponding author.

## Supplementary Materials

The following supporting information can be downloaded at https://www.mdpi.com/article/10.3390/biom16081198/s1: Supplementary Material S1 includes Figure S1: Assessment of MTAP status and PRMT5 inhibition in *MTAP*-isogenic cells; Figure S2: Generation and characterization of MRTX1719-resistant NSCLC cells in vitro; Figure S3: SDMA suppression following MRTX1719 and EPZ015666 treatment in paired DMSO and MRTXR cells; and Figure S4: High-throughput drug screen and MEK inhibitor sensitivity in MRTX1719-resistant NSCLC cells. Supplementary Material S2 includes the original Western blot images. Supplementary Material S3 includes Supplementary Tables S1–S6, which provide detailed information on the generated *MTAP*-isogenic cells, drug screening results, RNA sequencing results, and KEGG enrichment results.
