# Supplementary material for: Acquired Resistance to the PRMT5 Inhibitor Confers Collateral Sensitivity to MEK Inhibition in MTAP-Null Non-Small Cell Lung Cancer
Source: Biomolecules. 2026 Aug 17;16(8):1198. doi: 10.3390/biom16081198 (PMC13510389; doi:10.3390/biom16081198)

Figure S1. Assessment of MTAP status and PRMT5 inhibition in *MTAP*-isogenic cells. **(A)** Immunoblot analysis of MTAP protein levels across a panel of NSCLC cell lines. **(B)** Immunoblot validation of MTAP expression in *MTAP*-isogenic murine and human cell lines. **(C)** Immunoblot analysis of SDMA levels in the A375 *MTAP*-isogenic cell pair following treatment with increasing concentrations of EPZ015666 or MRTX1719.

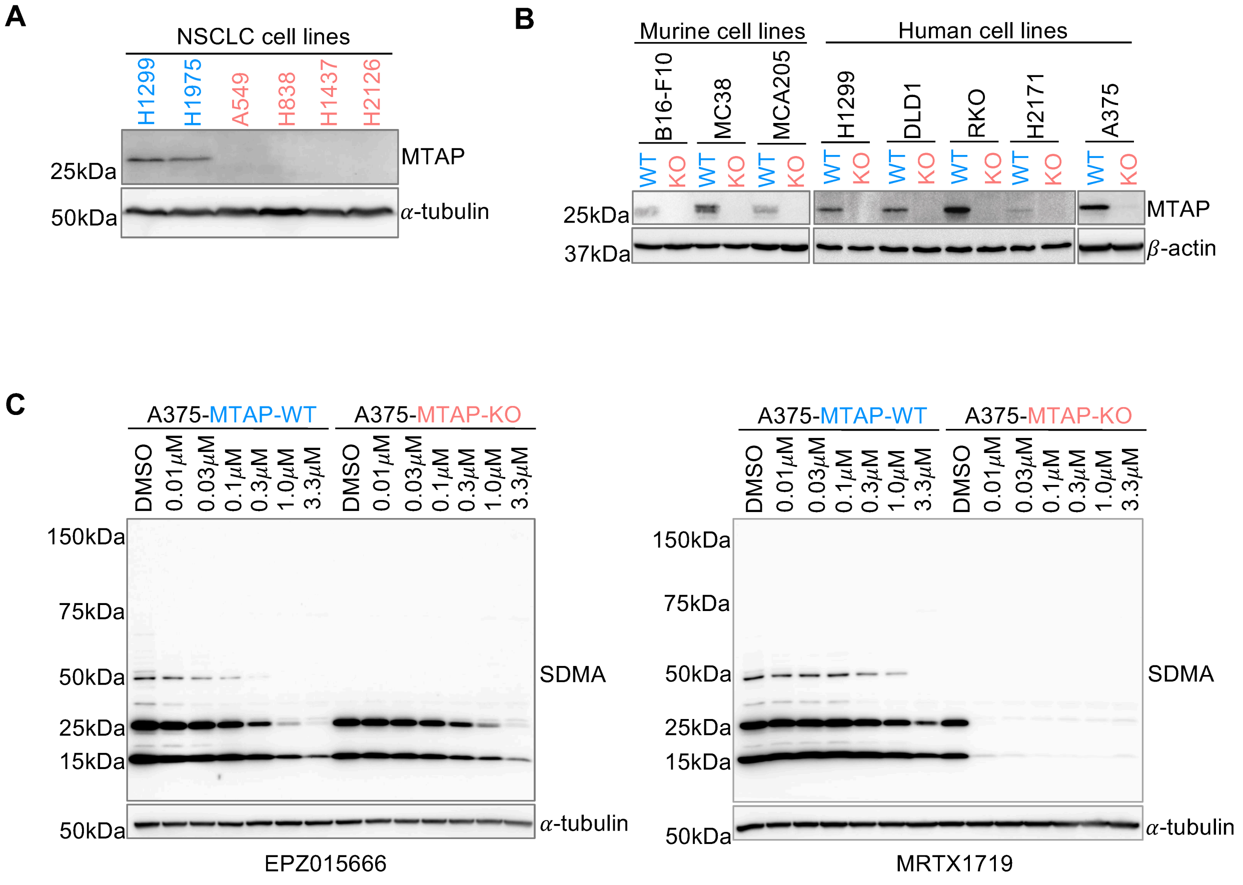

Figure S2. Generation and characterization of MRTX1719-resistant NSCLC cells in vitro. **(A)** Comparison of viable cell numbers in MRTX1719-treated and paired DMSO-treated cultures during the 25-week resistance-generation period. The table shows the actual MRTX1719 concentrations administered to each cell line at the indicated treatment stages. Viable cell number was calculated as the number of MRTX1719-treated cells relative to the corresponding DMSO-treated control cells. Cells were counted on day 7 of each treatment cycle and reseeded at their respective initial seeding densities; therefore, the plotted values represent weekly relative cell growth rather than cumulative cell numbers. Arrows indicate transitions between treatment stages. **(B)** Mean cell size of paired DMSO-treated and MRTX1719-treated cells measured using an automated cell counter at the indicated treatment weeks. **(C)** MRTX1719 dose–response assays in A549 paired DMSO-treated control (DMSO) and MRTX1719-resistant (MRTXR) cells after 1 month of drug withdrawal following prolonged MRTX1719 treatment.

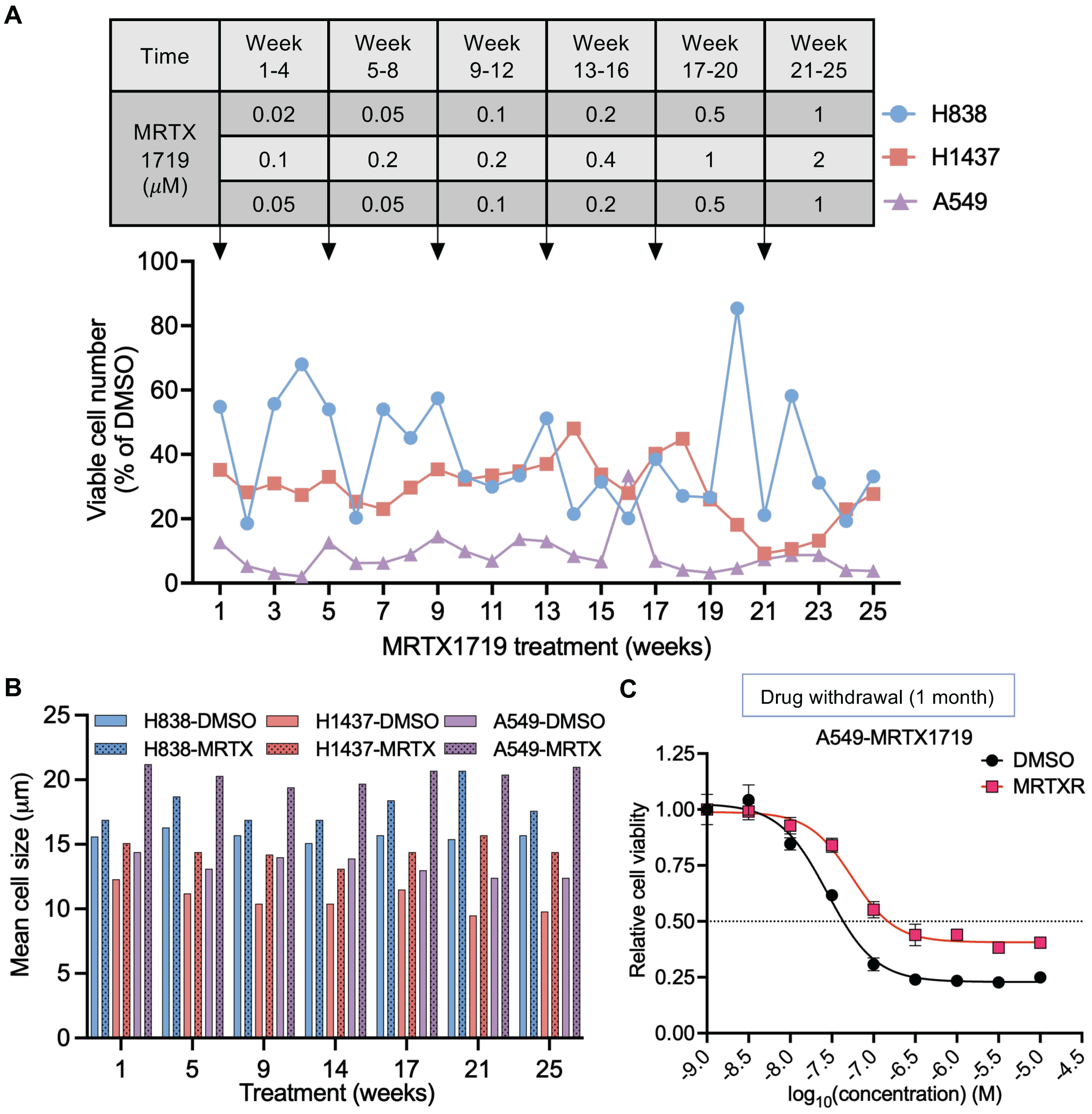

Figure S3. SDMA suppression following MRTX1719 and EPZ015666 treatment in paired DMSO and MRTXR cells. **(A)** Immunoblot analysis of time-dependent SDMA suppression in paired DMSO and MRTXR cells following treatment with 0.1 μM MRTX1719 for the indicated durations. **(B)** Immunoblot analysis of SDMA in paired DMSO and MRTXR cells following treatment with the indicated concentrations of EPZ015666 for 5 days.

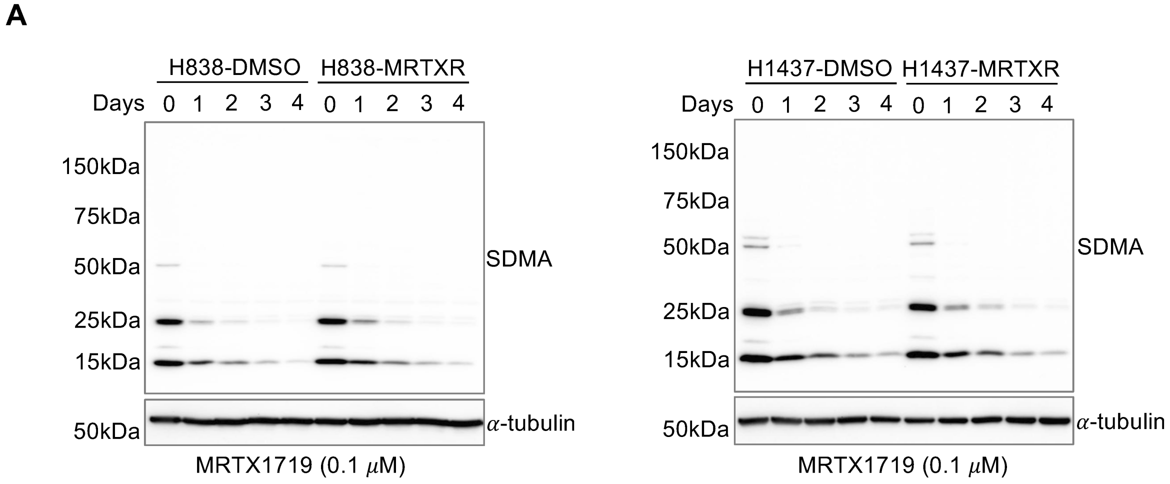

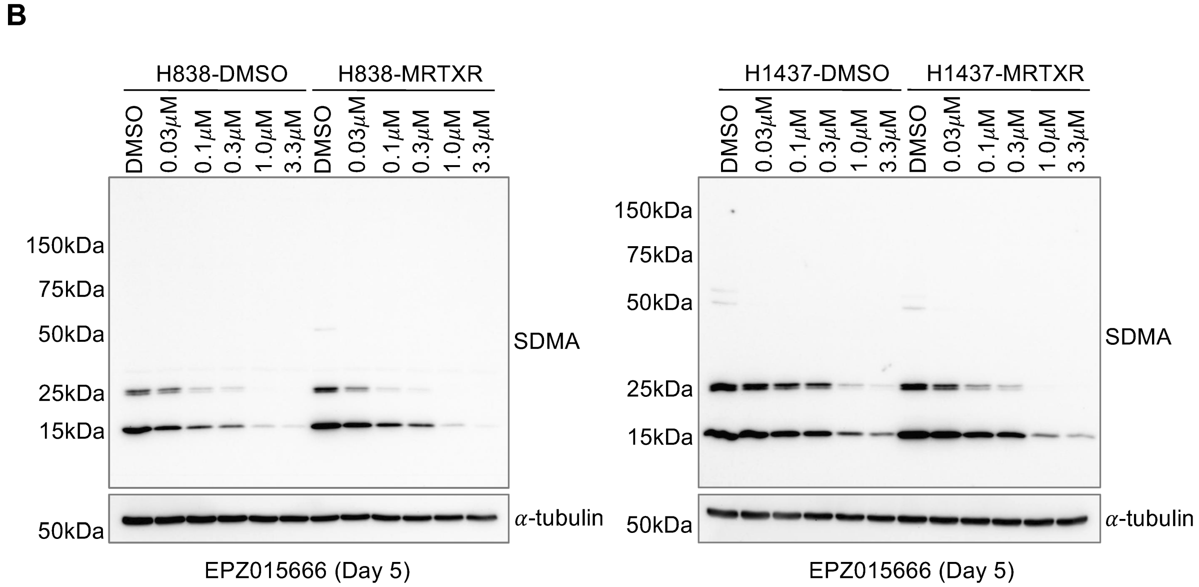

Figure S4. High-throughput drug screen and MEK inhibitor sensitivity in MRTX1719-resistant NSCLC cells. **(A)** Composition of the compound library used for drug screen, including SGC epigenetic compounds, FDA-approved oncology drugs, and TargetMol epigenetic inhibitors. **(B)** IC50 values of MRTX1719 and anisomycin in DMSO and MRTXR cells. Anisomycin was included as a nonselective control in the drug screen. **(C)** Dose–response curves of DMSO and MRTXR cells treated with the MEK inhibitor selumetinib. Data are presented as mean ± SD. **(D)** Synergy heatmaps of MRTX1719 and selumetinib in DMSO and MRTXR cells. Synergy mean scores were calculated using the Bliss model with the SynergyFinder+ tool.

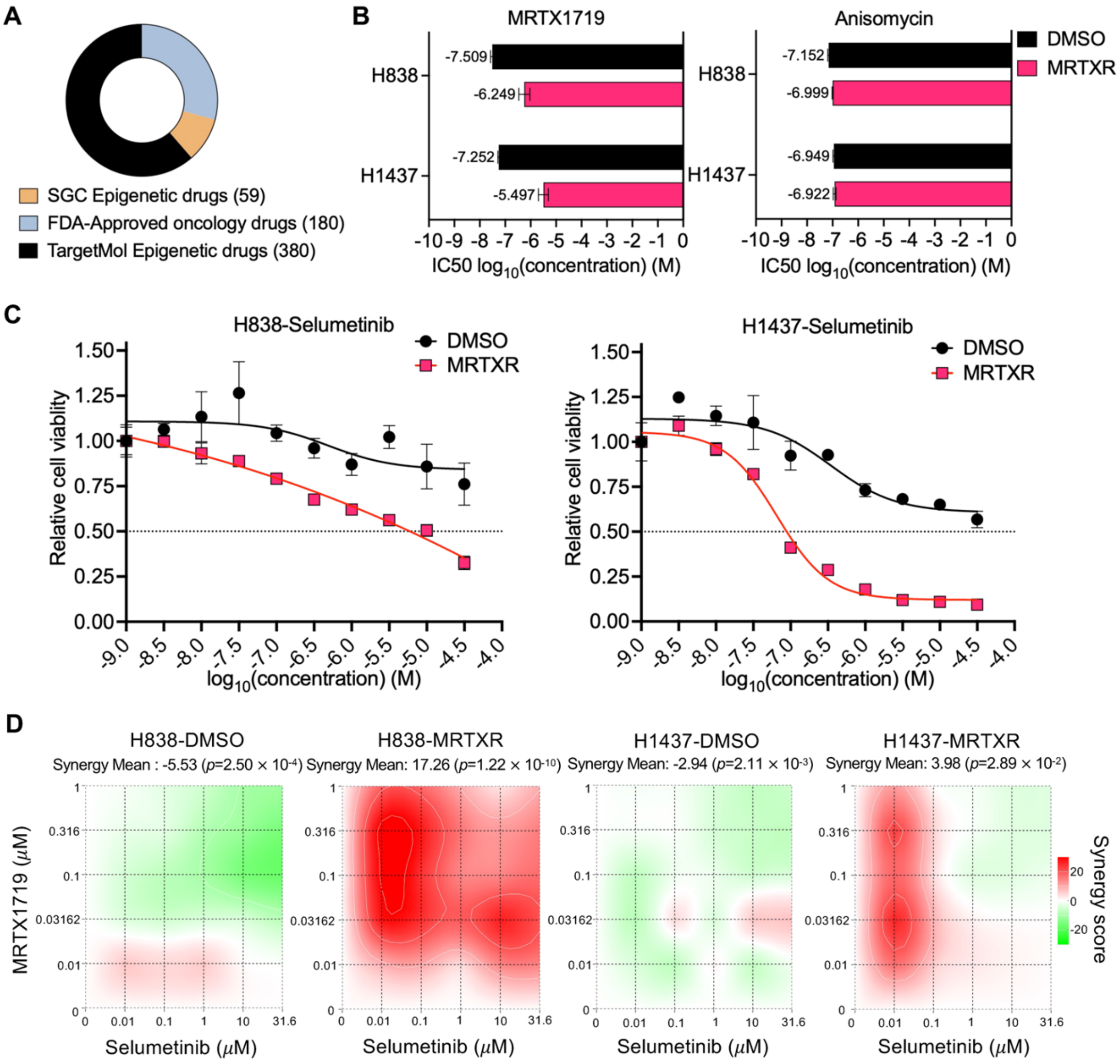

Supplement: Supplementary file 1 [file biomolecules-16-01198-s001.zip › Supplementary Material S1.pdf]
