# Supplementary material for: Acquired Resistance to the PRMT5 Inhibitor Confers Collateral Sensitivity to MEK Inhibition in MTAP-Null Non-Small Cell Lung Cancer
Source: Biomolecules. 2026 Aug 17;16(8):1198. doi: 10.3390/biom16081198 (PMC13510389; doi:10.3390/biom16081198)

Original Western blot Images

Figure 1C

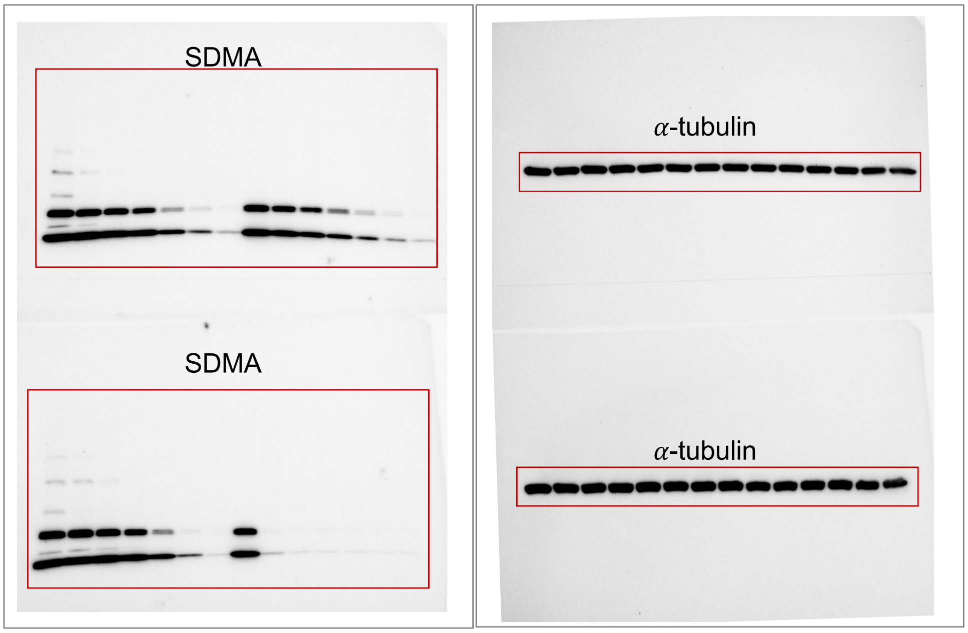

Figure 3A

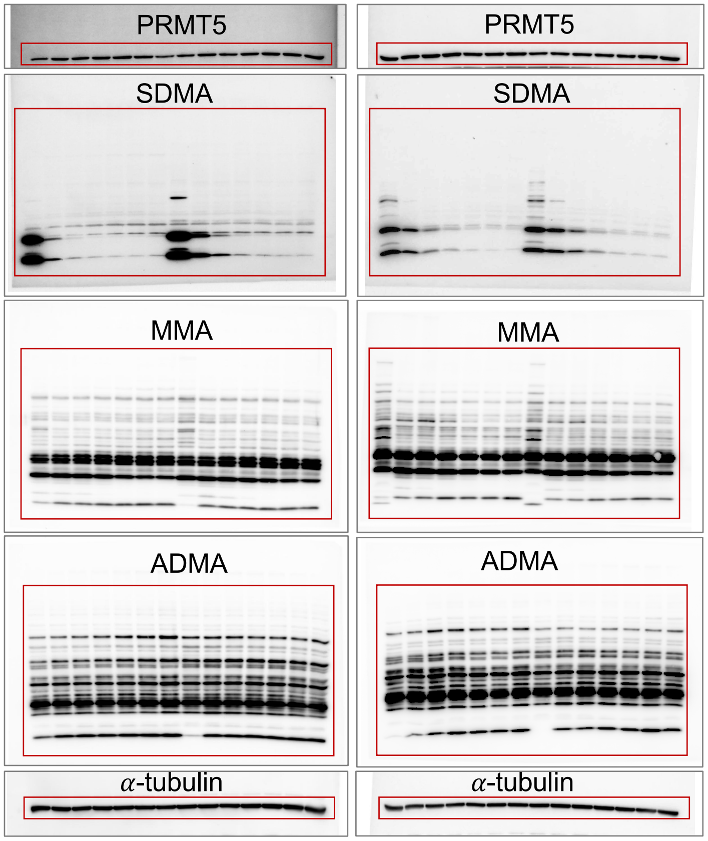

Figure 4D

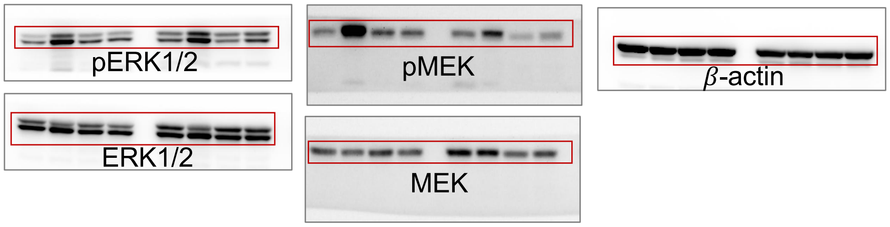

Figure S1A

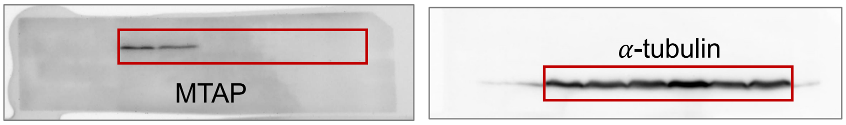

Figure S1B

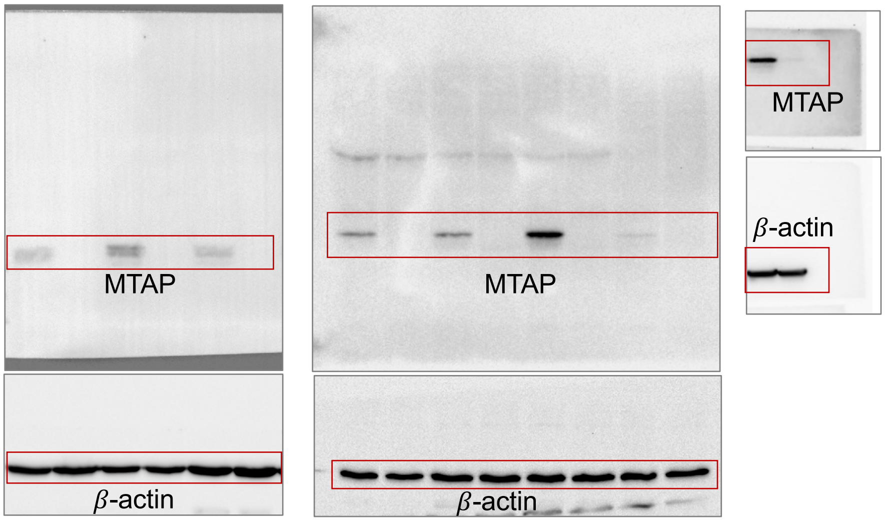

Figure S1C

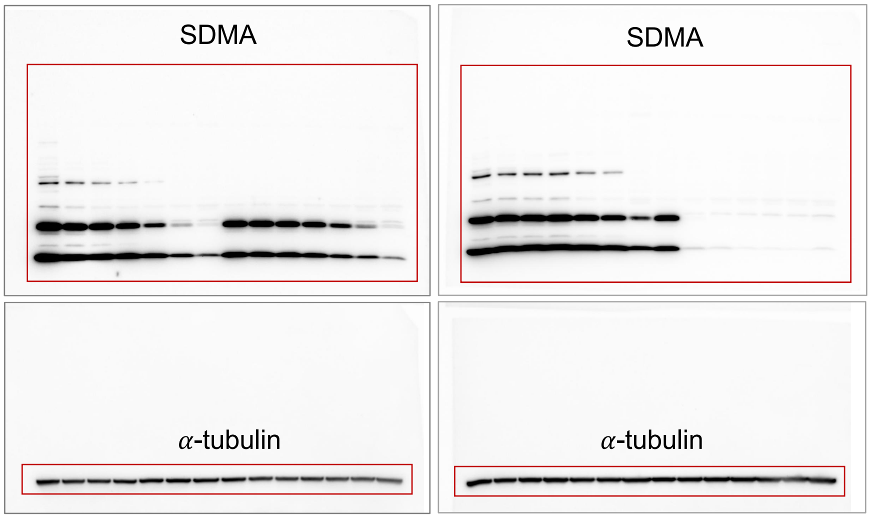

Figure S3A

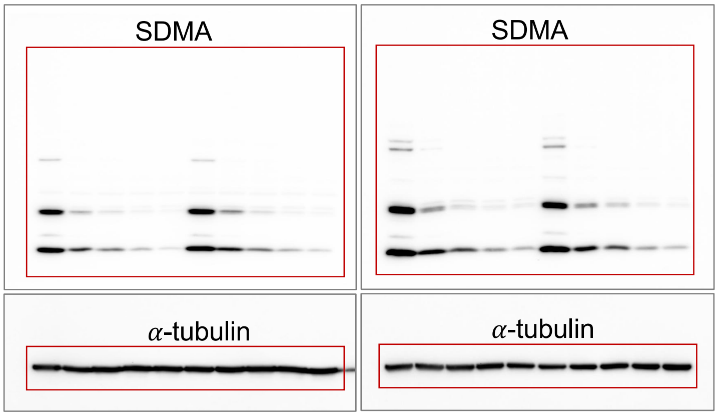

Figure S3B

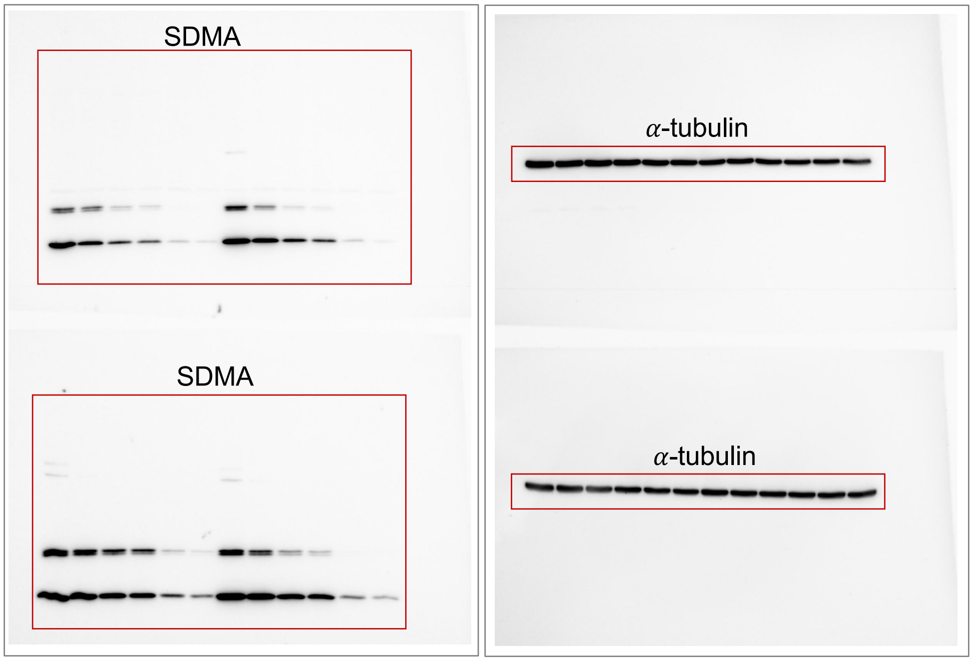

Supplement: Supplementary file 1 [file biomolecules-16-01198-s001.zip › Supplementary Material S2.pdf]
